# Specific type of childhood trauma and borderline personality disorder in Chinese patients

**DOI:** 10.3389/fpsyt.2022.936739

**Published:** 2022-07-26

**Authors:** Yanru Wu, Yuchen Zheng, Jijun Wang, Tianhong Zhang

**Affiliations:** Shanghai Mental Health Center, Shanghai Jiao Tong University School of Medicine, Shanghai, China

**Keywords:** borderline personality disorder (BPD), childhood trauma, childhood maltreatment, China, co-morbidity

## Abstract

**Background:**

Childhood maltreatment (CM) is a known risk factor for the development of mental disorders. An extensive body of literature about CM and mental health has been developed in wealthy countries, but information about this connection is lacking in developing countries including China.

**Aims:**

To explore the possible relationship between specific types of CM and borderline personality disorder (BPD) in patients with mental disorders in China.

**Methods:**

A survey was conducted in 2006, involving over 3,402 Chinese individuals aged 18–60 years who were randomly selected from the outpatients in the Shanghai Mental Health Center. The patients were screened with the Personality Diagnostic Questionnaire and CM was assessed using the Childhood Trauma Questionnaire. The final sample comprised 178 patients with BPD, 178 patients with other personality disorders (PDs), and 178 patients without PDs.

**Results:**

In Chinese patients, compared to other PDs, patients with BPDs are more likely to have experienced CM. Emotional maltreatment (emotional abuse and neglect) was the strongest predictor of BPD. Female gender and sexual abuse are significant predictors of the self-harm/suicidal risk of BPD patients.

**Conclusion:**

This is a pioneering study conducted on a large set of Chinese clinical samples with paired controls to establish and compare the associations between specific CM and BPD. Further studies in this field are necessary to elucidate the mechanism of how various types of childhood trauma have influenced PDs.

## Introduction

Borderline personality disorder (BPD) is a complex and serious mental disorder, characterized by a pervasive pattern of instability in affect regulation, impulse control, interpersonal relationships, and self-image (1, 2). As the most common personality disorder (PD) in clinical populations, BPD is associated with severe functional impairment (3–5), as well as significant individual and societal costs (6–8). For better prevention and treatment of BPD, recently more attention is focused on the risk factors of this disease, including the influence of early experience, especially childhood maltreatment (CM).

Childhood maltreatment is related with varies mental diseases in adulthood (9, 10), including psychosis (11), mood disorders (12), anxiety disorders (13), and PDs (14, 15). To date, many empirical studies have explored the connection between different types of CM (i.e., emotional abuse, physical abuse, sexual abuse, emotional neglect, and physical neglect) and BPD (9, 14, 16–24). The results generally supported that CM is a prominent contributor to BPD risk. However, the effect of specific types of CM on BPD is not clear because the related findings lack consensus. Some studies claimed that certain types of CM are uniquely associated with BPD, while others have shown their relationship with different PDs (9, 14, 16–24). Such varies among findings might be caused by a complex of factors, such as the method of sample collection, the limitation of retrospective research, and the influence of different socio-cultural contexts.

Moreover, there is a relative dearth of information in the current literature about Chinese patients with BPD. Till now, there are few Chinese studies accounting for CM and BPD (25, 26). Most of them were settled among Chinese undergraduate students (25), with the limited representation of the sample. Huang et al. conducted a study among 382 outpatients in 2007 and applied the Childhood Experiences of Care and Abuse Questionnaire (CECA-Q) as the method for evaluating the existence of CM (26). As the most used method in similar studies is the Child Trauma Questionnaire (CTQ) (27, 28), the results of Huang et al. are not readily comparable with other studies. Considering the strong difference between Chinese and Western culture, as well as the rapid changes in Chinese society (including the proceeding of westernization and modernization in the latest decades), the study of Chinese samples could not only provide contextually grounded clinical work with BPD patients but also provide one-to-one comparable data to further understand the interaction of CM and BPD.

Our previous study examined the clinical features and Axis I and II comorbidity of BPD and proved that childhood trauma has the most significant impact on Cluster-B PD (29). The aim of this study is to find out which types of CM were more prevalent in BPD samples compared with non-clinical and other PDs, and to compare the correlation between CM and BPD in Chinese patients with that in patients in western countries.

We hypothesis that: (1) patients with BPD would display more CM than the other two groups, (2) some special subtypes of CM would contribute to BPD more than other subtypes, and (3) some special subtypes of CM would contribute to the severity of BPD patients.

## Materials and methods

### Sample

The epidemiologic survey was conducted in 2006 at Shanghai Mental Health Center (SMHC), one of the largest medical health service settings in China (30–35). In former studies (30, 31, 34, 35), the participants were sampled from the outpatients randomly in the psycho-counseling clinics and psychiatric clinics at SMHC. In these studies, a total of 3,402 random outpatients were enrolled between May 2006 and October 2006. The information about the PD of each individual was collected by sending a self-report questionnaire to him/her. Exclusion criteria were set to ensure that all included individuals were in a stable state and have a certain degree of insight. Those with serious or acute psychotic symptoms were excluded. Only 3,075 subjects were included in the study. The response rate was 90.4% overall. More details of the exclusion criteria could be viewed in previous publications of ours (29, 30, 34).

### Measures

#### General questionnaire

The general questionnaire collected the following data: (a) demographics; (b) family and social background; and (c) physical and mental health conditions.

#### Assessment of personality disorders

##### The personality diagnostic questionnaire 4th edition plus (PDQ-4+)

A concisely structured self-report questionnaire, as described in our previous publications (29–31, 34). The questionnaire screens for 12 Axis II DSM-IV Personality Disorders using 107 true-false questions. The goal of PDQ-4+ is to distinguish individuals with and without characteristics associated with PD (36–38). The PDQ-4+ has high sensitivity (0.89), with acceptable specificity (0.65). It was used to screen DSM-IV PD in Chinese psychiatric patients (34, 39) as well as college student populations (40). The high test-retest reliability value (0.92) amongst the Chinese population indicates that the results yielded by this questionnaire are reliable.

##### The structured clinical interview for DSM-IV Axis II

A semi-structured PD diagnosis clinical interview. DSM-IV criteria were used for the classification of PDs in structured clinical interview for DSM-IV Axis II (SCID-II). The classification items include Cluster A PD (Paranoid, Schizoid, Schizotypal PD), Cluster B PD (Histrionic, Narcissistic, Borderline, Antisocial PD), Cluster C PD (Avoidant, Dependent, Obsessive-compulsive PD), Passive-aggressive PD and Depressive PD (in the appendix of DSM-IV). The results of SCID-II have high consistency (0.90) with clinical diagnosis. The test-retest reliability was good (0.70) (41).

#### Assessment of childhood maltreatment

##### The Child Trauma Questionnaire

A structured self-report questionnaire. CTQ uses 28 questions to assess CM (27). The CM is categorized in five subscales, namely, emotional abuse, physical abuse, sexual abuse, emotional neglect, and physical neglect. CTQ also provides a quantitative index of the severity of each subscale, each ranging from 5 (low level of CM) to 25 (high level of CM). In 1998, Bernstein et al. reported reliability coefficients (0.55) of CTQ’s 5 scales as satisfactory, with a particularly strong Cronbach’s α coefficient of 0.92 for the subscale of sexual abuse (42).

### Procedures

This study was approved by the Research Ethics Committee at SMHC in 2006. Enrolled individuals were selected with a two-stage procedure. In the first stage, 3,402 individuals were randomly selected from the psycho-counseling and psychiatric clinics in SMHC. The enrolled individuals were asked to take a general questionnaire and PDQ-4+. The data were reviewed by trained nurses, to ensure that each submitted questionnaire was completely answered. The general questionnaire was used to collect basic information about the participants, as described in section “General Questionnaire.” Then a trained psychiatrist reviewed the PDQ-4+ and screened for PDs. In total, 2,570 out of the 3,075 eligible participants met the criteria for PD in DSM-IV. These 2,570 participants with PD were then recruited and entered the second stage of the study.

**FIGURE 1:**
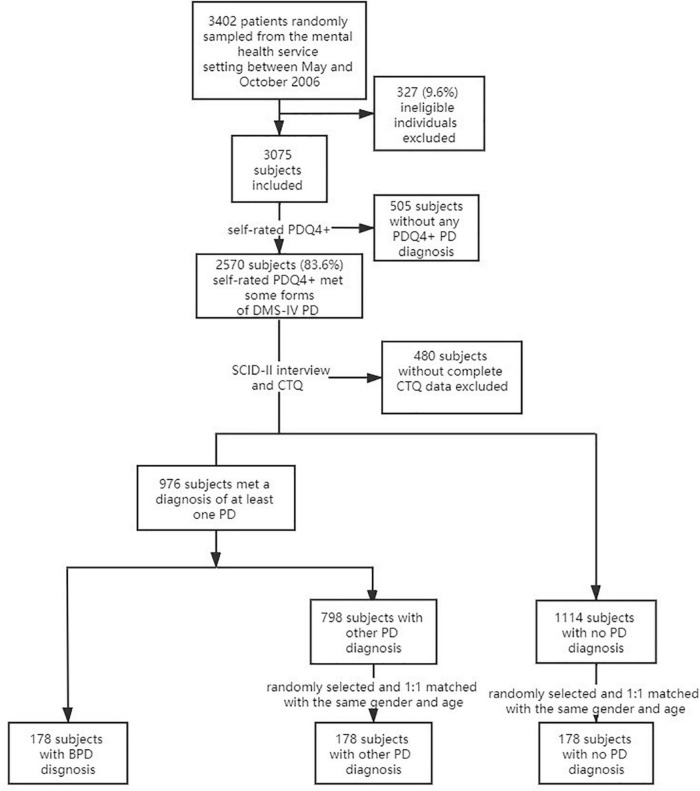
The flow of subject selection and data extraction.

In the second stage, SCID-II clinical were performed on the patients by two senior psychiatrists trained for 2 weeks by the research team members. Prior to the interview, the psychiatrists were concealed from the PDQ-4+ test results and clinical diagnosis results of the patients to reduce the subjective deviation. The two psychiatrists rated 30 patient interviews independently, and the Kappa value of reliability for any PD was 0.82, indicating good inter-rater reliability. All 2570 patients have taken the SCID interview and were asked to complete CTQ. A total of 484 patients were excluded due to incomplete CTQ data (81.1% responding rate). Of the 2086 included patients, 178 were diagnosed with BPD with SCID-II. For the control group, 178 patients who were diagnosed with other personality disorders were randomly selected from the sample of 798 subjects with other kinds of PD diagnoses. This group was dubbed as “other PD group.” Another 178 patients without PD diagnosis were randomly selected from the sample of 1,110 subjects with no PD diagnosis. This group was dubbed as “no PD group.” The two control groups were matched with the BPD group in gender and age (see Figure 1).

### Statistical analysis

SPSS 22.0 (SPSS Inc., Chicago, IL, United States) was applied to analyze participants’ demographic and clinical data. Patients in the BPD group and two control groups were evaluated by PDQ-4+ and SCID-II for PDs. Frequencies and 95% CI (95% CI) were calculated separately by cluster and specific PD. Chi-squared tests were used to compare the demographic and clinical profiles such as gender, marriage state, raising environment, parents’ marriage state, family history of mental disease, physical comorbidity, and self-reported character between different PD groups.

Two-tailed *t*-tests were used to compare the age, length of illness, education, and the average score of CTQ subscales between different PD groups. Means (M) and SD were calculated for those continuously distributed variables.

Odds ratios (OR) were generated to assess associations of PDs with demographic and clinical profiles such as age, gender, education and marriage state, parents’ marriage state, physical and mental comorbidity, and self-reported characteristics.

Logistic stepwise regression was performed using different PD groups (BPD vs. Other PD, BPD vs. no PD, and Other PD vs. no PD) as dependent variables, with the average score of CTQ subscales as independent variables.

All statistical differences were considered significant at *p* < 0.05.

## Results

### Demographics and clinical characteristics

In this observational study, there were 48 males and 130 females in each paired group (Table 1). Compared with the two control groups, significantly more individuals in the BPD group were raised without both parents, had a parental divorce, and were diagnosed with mood disorders. These groups were similar in sex or age, length of illness (mental disease), education, physical comorbidity, family history of mental disease, or personal character. There were no significant differences in any demographic characteristics between the two control groups.

**TABLE 1:** Clinical and socio-demographic characteristics of BPD, other PD, and no PD group.

	BPD (*n* = 178)		Other PD (*n* = 178)		No PD (*n* = 178)		BPD vs. Other PD		Other PD vs. No PD		BPD vs. No PD	
	N	%	N	%	N	%	X^2^	*P*	X^2^	*P*	X^2^	*P*
Gender							0.000	1.000	0.000	1.000	0.000	1.000
Male	48	27.0	48	27.0	48	27.0						
Female	130	73.0	130	73.0	130	73.0						
Marital Status (Married)	53	29.8	45	25.3	52	29.2	0.901	0.203	0.694	0.475	0.014	1.000
Raised by both parents	140	78.7	154	86.5	157	88.2	3.828	0.034*	0.229	0.750	5.871	0.022*
Parental divorce	31	17.4	16	9.0	14	7.9	5.515	0.014*	0.134	0.849	7.245	0.010*
Family history of mental disease	22	12.4	19	10.7	16	9.0	2.408	0.740	0.285	0.722	1.061	0.391
Physical comorbidity	39	22.7	24	14.2	21	12.4	3.962	0.051	0.273	0.634	6.296	0.015
Mental comorbidity												
Psychotic disorder	20	11.2	55	30.9	72	40.4	21.962	< 0.001**	4.398	0.111	39.634	< 0.001**
Mood disorder	86	48.3	53	29.8	34	19.1	12.853	< 0.001**	5.491	0.019*	33.991	< 0.001**
Anxiety disorder	22	12.4	43	24.2	29	16.3	8.300	0.004**	3.412	0.065	1.121	0.290
Character							2.511	0.285	3.827	0.148	0.269	0.874
Introversion	61	34.3	75	42.1	59	33.1						
Middle type	81	45.5	74	41.6	79	44.4						
Extroversion	36	20.2	29	16.3	40	21.3						

	**Mean**	**SD**	**Mean**	**SD**	**Mean**	**SD**	**F/Z**	***P***	**F/Z**	***P***	**F/Z**	***P***

Age (years)	26.71	6.168	26.74	6.219	26.98	6.135	0.233	0.630	0.267	0.606	0.001	0.945
Length of illness (months)	42.50	55.268	40.35	47.803	38.96	51.293	0.061	0.805	0.051	0.821	0.182	0.670
Education (years)	13.264	2.847	14.297	2.671	13.792	2.810	2.449	0.118	1.776	0.184	0.062	0.804
CTQ score												
Emotional abuse	10.10	4.194	8.18	2.796	7.26	2.246	33.252	< 0.001**	4.473	0.035*	63.010	< 0.001**
Physical abuse	7.47	3.364	6.04	1.738	6.29	1.660	54.094	< 0.001**	0.876	0.350	49.932	< 0.001**
Sexual abuse	6.49	2.318	6.13	1.915	5.94	1.536	8.483	0.004**	1.268	0.261	19.203	< 0.001**
Emotional neglect	14.79	5.490	11.49	4.203	10.62	4.032	16.817	< 0.001**	0.663	0.416	23.264	< 0.001**
Physical neglect	9.73	3.648	8.09	2.667	8.26	2.756	17.293	< 0.001**	0.062	0.804	14.704	< 0.001**
CTQ (total)	48.52	13.307	39.90	8.240	38.07	6.905	37.028	< 0.001**	3.504	0.062	59.526	< 0.001**

Age grouped by median age of the sample. Levene’s Test for Equality of Variances is significant; *p < 0.05; **p < 0.01. CTQ, The Childhood Trauma Questionnaire.

### The comorbidity of personality disorder diagnoses between borderline personality disorder group and other personality disorders group

**FIGURE 2:**
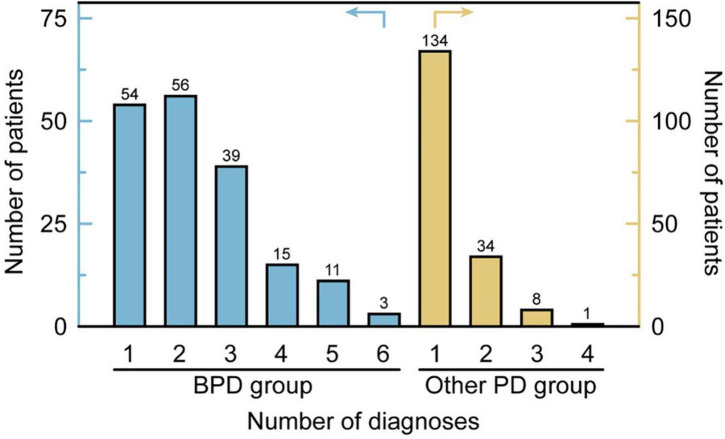
The number of comorbidities of PDs in the BPD group and other PD groups.

All PD patients were divided into subgroups with the overall number of PD diagnoses they have (minimum one, maximum six). The histogram (Figure 2) presents the distribution of patients in the subgroups. In total 44 patients (24.7%) in the other PD group has more than one PD diagnosis, while 124 patients (69.7%) in the BPD group have more than one PD diagnosis. In the BPD group, 68 patients (38.2%) have at least 3 PD diagnoses. There was only one patient (0.6%) who had more than 3 PD diagnoses in other PD groups.

### The results of Child Trauma Questionnaire among different groups

**FIGURE 3:**
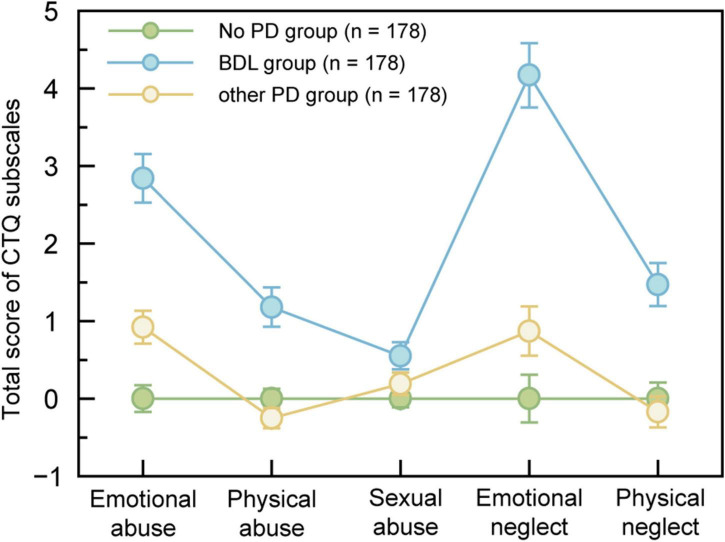
Childhood trauma profile by different PD groups. Marginal means from mixed models were standardized with control means (SDs) to convert to z-score. Error bars denote SEMs within groups.

Child Trauma Questionnaire in the three groups shows distinct results (Table 1). The variants were transformed into *z*-score (Figure 3). Compared with the two control groups, the BPD group has the highest scores in each one of the subscales. The two subscales, emotional neglect, and emotional abuse, display the most significant difference between BPD groups and control groups. This indicated that emotional maltreatment (emotional abuse and neglect) was the strongest predictor of BPD.

Stepwise regression was employed to identify the risk factors of BPD related to childhood traumatic events. Logistic regression (forward stepwise) analyses were performed. BPD group, other PDs group, and no PD group were used as dependent variables. Different childhood trauma (emotional abuse, physical abuse, sexual abuse, emotional neglect, and physical neglect) were used as independent variables (Table 2). The results indicated that compared with the no PD group, emotional abuse and emotional neglect were significant predictors of BPD, and emotional abuse was a significant predictor of other PDs. The significant predictors of BPD from other PDs were physical abuse and emotional neglect.

**TABLE 2:** Forward stepwise logistic regression for risk factors predicting the clinical diagnoses of different PD groups.

	Variable	beta	S.E.	OR	95% CI	χ^2^ statistic	*P*-value
BPDs vs. Other PDs	Physical abuse	0.175	0.054	1.191	1.073–1.323	10.669	0.001
	Emotional neglect	0.114	0.024	1.120	1.068–1.175	21.670	< 0.001
	Constant	–2.631	0.436	0.072	–	36.447	0.072
BPDs vs. No PD	Emotional Abuse	0.186	0.047	1.204	1.099–1.320	15.805	< 0.001
	Emotional neglect	0.116	0.029	1.120	1.062–1.189	16.352	< 0.001
	Constant	–2.997	0.414	0.072	–	52.510	< 0.001
Other PDs vs. No PD	Emotional Abuse	0.146	0.045	1.157	0.791–0.944	10.541	0.001
	Constant	–1.101	0360	0.333	–	9.361	0.002

### The difference in demographics and Child Trauma Questionnaire between patients with and without self-harm/suicide in the borderline personality disorder group and other personality disorders group

All PD patients were divided into subgroups according to that whether they had a medical history of self-harm behavior/suicide or not. The existence of self-harm behavior/suicide was evaluated by the 45th item in PDQ–4+. As shown in Table 3, in the BPD group 117 patients had self-harm behavior/suicide, while in other PDs group 62 patients had such behavior. In the BPD group, the self-harm behavior/suicide subgroup showed significant differences in gender and the score of sexual abuse. In other PDs groups, the self-harm behavior/suicide subgroup showed significant differences in marital status, age, and sexual abuse.

**TABLE 3:** Different PD groups with self-harm/suicidal behaviors vs. without self-harm/suicidal behaviors.

	BPD (n = 178)			Other PD group (*n* = 178)		
	PDQ45 + N (%)	PDQ45−	*t*-value	PDQ45+	PDQ45−	*t*-value
Number	117	61	/	62	116	/
Gender (male)	26 (22.2)	22 (36.1)	3.902*	14 (22.6)	34 (29.3)	0.929
Marriage (married)	46 (39.3)	25 (41.0)	0.003	14 (22.6)	41 (35.3)	4.218*
Age	26.44 (5.802)	27.23 (6.837)	1.046	25.31 (5.259)	27.50 (6.571)	4.712*
Emotional abuse	10.52 (4.406)	9.29 (3.639)	3.299	9.19 (2.963)	7.63 (2.549)	1.501
Physical abuse	7.51 (3.528)	7.39 (3.057)	0.801	6.38 (2.091)	5.86 (1.498)	2.854
Sexual abuse	6.69 (2.458)	6.12 (1.984)	4.927*	6.52 (2.560)	5.91 (1.411)	8.040*
Emotional neglect	14.86 (5.410)	14.65 (5.686)	1.316	11.95 (4.630)	11.24 (3.962)	1.418
Physical neglect	9.68 (3.578)	9.83 (3.805)	1.832	8.55 (2.708)	7.84 (2.623)	0.014

PDQ45±: The result of the 45th item in the Personality Diagnostic Questionnaire 4th edition plus was positive/negative.

## Discussion

### Major findings

The primary aim of the present study was to examine the relationship between CM and BPD, as well as the difference comparing BPD with other PDs and no PD patients among the mental disorders population. We noted three key findings. First, patients with BPD reported the most severe CM and comorbidities of PDs among the three groups. Second, BPD and other PDs are associated with different types of CM. Third, the self-harm behavior/suicide in BPD and other PDs are associated with different demographical factors and the same CM (sexual abuse). These findings indicated that there may be different interactive patterns between CM and BPD compared with other PDs. To the best of our knowledge, this is among the pioneering studies conducted in a large paired Chinese clinical sample to establish and compare the associations between specific CM and BPD.

### Borderline personality disorder with childhood maltreatments and comorbidities of personality disorders

According to previous studies, a large number of BPD patients reported having experienced CM (9, 14, 16–24, 43, 44), and different types of CM often co-occur and psychiatric symptom severity increase with the number and severity of experienced maltreatment types (45, 46). The results of our studies supported these facts and provided extra evidence by proving that patients with BPD present severer clinical profiles (i.e., the co-morbidity of PDs) and reported a higher levels of CM than other PDs. As we have discussed in our previous report (29), the broad range and high severity of CM related to BPD might be caused by insecure attachment style, which is a common result of CM. This is in accordance with our finding, that significantly more BPDs patients were raised without both parents or had a parental divorce when compared with the other two groups. The relationship between insecure attachment style, CM and BPD has been wildly discussed (47, 48), but there is a lack of clinical evidence in the Chinese population. More research about the relationship between attachment style, CM and BPD in the Chinese population is needed.

### Specific childhood maltreatments associated with borderline personality disorder and other personality disorders

Regarding the relationship between BPD and self-reported CM, this research has shown that greater levels of emotional neglect and emotional abuse are most significantly related to BPD, and physical abuse and emotional neglect are two factors that differ BPD from other PDs. Such results are consistent with previous studies, including one of the latest meta-analyses, that emotional maltreatment (emotional abuse and neglect) was the strongest predictor of BPD and symptom severity (9, 22, 49).

The negative results in our studies are also in accordance with previous studies conducted in western countries. Many previous studies have discussed the possible correlation between sexual abuse and BPD traits (19, 50, 51), while the latest meta-analysis has presented more negative evidence about sexual abuse as a causal factor of BPD (49). In our study, no significant difference in sexual abuse was found among groups. This result might be related to the low frequency [12.5% in our study vs. 30–45% in western countries (52)] of sexual abuse reported in our survey, which might be related to the fact that comparably less cases of childhood sexual abuse were reported in Chinese society than western countries (53). Although cultural differences, sample characteristics and methodological aspects (54) should be taken into consideration while explaining the results, the types of CM in Chinese BPD patients are much the same as in other western countries.

### Different demographical factors and childhood maltreatments associated with the self-harm/suicidal behavior in borderline personality disorder and other personality disorders

More samples with self-harm/suicidal behaviors were found in the BPD group, which was also reported in studies conducted in western countries (55–57). In this study, two risk factors were found to be significant predictors of the self-harm/suicidal risk of BPD patients, namely, female gender and sexual abuse in childhood. The significant predictors of the self-harm/suicidal risk of other PD are marital status, age, and sexual abuse in childhood. Although sexual abuse was not a predictor of BPD from other PDs, it serves as a necessary factor that might promote mood instability and affect impulse control, leading to self-harm/suicidal behavior. Such result was consistent with the result of Brodsky et al., who in their study found that sexual abuse was implicated as a predictor of suicidal attempts in cross-sectional studies (58). In other PDs groups, the age distribution has shown that the older age, the less likelihood of self-harm/suicidal attempts. It was unexpected that no significant difference with age between subgroups in BPD patients, although there was a weak connection between higher risk and younger age. Such a result might be related to the fact that the non-suicidal self-injury (NSSI) and deliberate self-harm (DSH) were not differentiated in this study, as well as other limitations of the measurement method. According to a previous study, there was an increase in rates of NSSI and DSH in adolescence and a decline in adulthood (59). There might be different self-harm/suicidal behaviors among various PDs in the Chinese population (60), which needs further studies.

### Application of this study

The findings in this research could lead to helpful applications in clinical practice and the prevention and treatment of BPD. In clinical practice, these findings suggest that the existence of a specific type of CM could be used as an indicator to help clinicians differentiate BPD from other PDs when the diagnosis is not so clear. Thus, to better understand and diagnose PD patients, clinicians must carefully evaluate CM during the interview. Besides, patient-reported multiple CM would remind the clinician to think about the existence of BPD. Furthermore, based on the relation between sexual abuse and suicidal behavior, the existence of sexual abuse could also be an indicator to remind clinicians to the risk on the patient. The result of this study could also be extended to the understanding of ICD-10 Emotionally Unstable Personality Disorder (EUPD), since BPD and EUPD may represent analogous diagnostic categories across classification systems (61).

Another application would be in the prevention and treatment of BPD. More attention should be paid to children suffering from maltreatment and related policy should be made to improve their environment. For those who have experienced trauma, supporting resources and timely follow-ups are necessary. Selective prevention strategies, such as family based risk prevention and resilience program, could be provided (62). When those victims present PD traits, some trauma-focused treatment techniques and interventions might be helpful. Currently, the mainstream psychotherapies for BPD, such as schema therapy, mentalization-based treatment, and reduced dialectical behavior therapy (63), are not specifically focused on the traumatic experience in the past. The findings of this study emphasized the necessity of trauma-oriented therapy in treating BPD patients.

### Limitations

The present study has several limitations. First, the assessment of self-harm/suicidal behavior was dichotomous rather than continuous, which therefore did not reflect the frequency and severity of those behaviors. Second, there was only one group of “other PDs” being compared with BPD, limited by the sample size. To acquire a deeper understanding of the mechanism of how specific CM interacts with BPD, more one-on-one comparisons need to be made between a specific type of PD and BPD. Third, our work did not establish the causal relationship between CM and the development of BPD, which is not within the scope of this cross-sectional study. Finally, because the survey was conducted in 2006 and in one hospital, the generalizability of the results of this study to the wider, non-treatment seeking population in the current era requires further examination.

## Conclusion

Our results have shown that: first, patients with BPD reported the most severe CM and comorbidities of PDs compared to other-PDs groups and no PD group. Second, emotional maltreatment (emotional abuse and neglect) was the strongest predictor of BPD. Third, female gender and sexual abuse are significant predictors of the self-harm/suicidal risk of BPD patients. Those factors have the potential to be targeted for clinical diagnosis, preventative intervention, and future research.

## Data availability statement

The datasets generated for this study are available upon request from the corresponding author.

## Ethics statement

The studies involving human participants were reviewed and approved by the Research Ethics Committee at Shanghai Mental Health Center. The patients/participants provided their written informed consent to participate in this study.

## Author contributions

YW and YZ designed and conducted this study, included the data analysis, and wrote the article. JW was responsible for recruiting, diagnosing, and classifying the patients. TZ revised the study design and the article. All authors contributed to the article and approved the submitted version.
